# Development of macrophage-associated genes prognostic signature predicts clinical outcome and immune infiltration for sepsis

**DOI:** 10.1038/s41598-024-51536-3

**Published:** 2024-01-23

**Authors:** Guangxin Ma, Xiaolin Wu, Cui Qi, Xiaoning Yu, Fengtao Zhang

**Affiliations:** 1 Department of Geriatric Medicine, Qilu Hospital, Cheeloo College of Medicine, Shandong University, Jinan, China; 2 Cancer Institute, Qingdao University, Qingdao, 266071 China; 3 Qingdao Women and Children’s Hospital, Qingdao, China; 4 Women and Children’s Hospital, Qingdao University, Qingdao, China; 5 Department of Anesthesia, Dezhou Municipal Hospital, Dezhou, China

**Keywords:** Computational biology and bioinformatics, Genetics, Immunology, Risk factors

## Abstract

Sepsis is a major global health problem, causing a significant burden of disease and death worldwide. Risk stratification of sepsis patients, identification of severe patients and timely initiation of treatment can effectively improve the prognosis of sepsis patients. We procured gene expression datasets for sepsis (GSE54514, GSE65682, GSE95233) from the Gene Expression Omnibus and performed normalization to mitigate batch effects. Subsequently, we applied weighted gene co-expression network analysis to categorize genes into modules that exhibit correlation with macrophage activity. To pinpoint macrophage-associated genes (MAAGs), we executed differential expression analysis and single sample gene set enrichment analysis. We then established a prognostic model derived from four MAAGs that were significantly differentially expressed. Functional enrichment analysis and immune infiltration assessments were instrumental in deciphering the biological mechanisms involved. Furthermore, we employed principal component analysis and conducted survival outcome analyses to delineate molecular subgroups within sepsis. Four novel MAAGs—CD160, CX3CR1, DENND2D, and FAM43A—were validated and used to create a prognostic model. Subgroup classification revealed distinct molecular profiles and a correlation with 28-day survival outcomes. The MAAGs risk score was developed through univariate Cox, LASSO, and multivariate Cox analyses to predict patient prognosis. Validation of the risk score upheld its prognostic significance. Functional enrichment implicated ribonucleoprotein complex biogenesis, mitochondrial matrix, and transcription coregulator activity in sepsis, with an immune infiltration analysis indicating an association between MAAGs risk score and immune cell populations. The four MAAGs exhibited strong diagnostic capabilities for sepsis. The research successfully developed a MAAG-based prognostic model for sepsis, demonstrating that such genes can significantly stratify risk and reflect immune status. Although in-depth mechanistic studies are needed, these findings propose novel targets for therapy and provide a foundation for future precise clinical sepsis management.

## Introduction

Sepsis represents a critical global health challenge, contributing significantly to the worldwide burden of morbidity and mortality. The Global Burden of Disease Study reports that in 2020, sepsis accounted for approximately 48.9 million incidences and 11 million fatalities across the globe^1^. Factors such as a burgeoning elderly population, the escalation of chronic disease prevalence, and a surge in antibiotic resistance have been implicated in the rising incidence of sepsis^2^. A 2020 Lancet study revealed an annual increase of 7.7% in the global incidence of sepsis from 1990 to 2017, underscoring the growing impact of this condition on individuals and societies at large^3^.

The clinical management of sepsis encompasses a range of critical interventions, one of which is risk stratification^2^. This process facilitates early detection and informs prognosis by evaluating the probability and severity of the condition, thereby enabling more rigorous monitoring and treatment. Such treatments may include prompt antibiotic therapy and aggressive fluid resuscitation^4^. However, the current approaches to risk stratification are not without their limitations, and advancing the timely initiation of treatment for patients with severe sepsis remains a pivotal challenge in enhancing patient outcomes^5^.

Macrophages play a pivotal role in orchestrating both the onset and the resolution of the immune response during sepsis^6^. In the initial phase of sepsis, these cells are primed to release pro-inflammatory cytokines, including tumor necrosis factor (TNF) and interleukin-1 (IL-1), which are crucial for recruiting and activating additional immune cells to combat the infection^7^. However, an overproduction of such cytokines can precipitate systemic inflammation and subsequent tissue harm, potentially leading to organ dysfunction and failure^8^. Furthermore, emerging evidence indicates that macrophages may exhibit a differential phenotype contingent on the sepsis stage; they typically display a pro-inflammatory profile in the early phase, but a more anti-inflammatory profile in the later stages^7,9^. Given the nuanced and dynamic nature of macrophage function in sepsis and the critical balance of pro- and anti-inflammatory responses, comprehensive research into the multifaceted roles of macrophages across different stages of sepsis is imperative.

In this investigation, we identified macrophage-associated genes (MAAGs) linked to sepsis prognostication. We selected four MAAGs—CD160, CX3CR1, DENND2D, and FAM43A—to construct a prognostic model for sepsis. The significant differential expression of these novel targets in sepsis was corroborated with clinical specimens. Moreover, we explored the potential mechanisms underlying the prognostic differences by conducting functional enrichment analyses and assessing immune infiltration. Our findings offer novel potential targets for sepsis treatment and contribute fresh insight into the involvement of macrophages in the condition.

## Materials and methods

### Dataset collection for sepsis and differential expression analysis

GSE54514, GSE65682 and GSE95233 datasets for healthy and sepsis samples were acquired from the Gene Expression Omnibus (GEO) database. The transcriptome data of three datasets were normalized to remove the batch effect via “sva” script. After the deletion of sepsis samples without 28 days survival time in GSE65682 (blood RNA was isolated at intensive-care unit (ICU) admission and throughout ICU length-of-stay), a total of 42 healthy samples and 479 sepsis samples were enrolled for the prognosis analysis. The differential expression analysis was performed with |fold change| ≥ 2 and p.adjust < 0.05.

### Identification of macrophage-associated genes (MAAGs)

On the basis of single sample gene set enrichment analysis (ssGSEA), the fraction of 23 immune cells were estimated. Then, “WCGNA” script was employed to explore the most pivotal gene module which related to immune cells. According to the most appropriate soft threshold, we established a scale-free network to construct the WGCNA. The samples in GSE65682 were clustered to exclude the abnormal samples and standards compliant samples were enrolled for the next analysis. Then, the dynamic tree cut the gene modules and merged into the final modules. Pearson correlation analysis was adopted to evaluate the relationship between each module. Finally, the correlation of each gene module and 23 immune cells was calculated to select the most characteristic module. According to the module-trait relationship, the black module was the most significant gene module associated with macrophage and the genes in black module was considered as macrophage associated genes (MAAGs).

### Generation of molecular subgroups for sepsis

On the basis of WGCNA and difference analysis, 480 intersection DE-MAAGs were obtained via “venn” script (Supplementary Table 1). “ConsensusClusterPlus” was adopted to cluster the sepsis in GSE65682 into different MAAGs molecular subgroups according to the optimal K of 2–9. Principal component analysis was carried out to clarify the distribution pattern of sepsis in MAAGs molecular subgroups via “ggplot2” script. Based on the corresponding clinical survival information for sepsis samples, “survival” script was performed to estimate the 28 days survival outcome for sepsis samples in MAAGs subgroup.

### Function enrichment analysis

With the selection threshold set at p.adjust < 0.05, the DEGs in MAAGs subgroups were calculated using “limma” script. “clusterProfiler” script was employed to enrich the DEGs into gene ontology (GO) terms. Moreover, “GSVA” script was carried out to assess the kyoto encyclopedia of genes and genomes (KEGG) terms of sepsis in MAAGs subgroups.

### Immune infiltration analysis

The single-sample Gene Set Enrichment Analysis (ssGSEA) method was used to assess the level of immune cell infiltration in sepsis samples across different MAAGs subgroups, utilizing the "GSVA" R package^10^. ssGSEA requires predefined gene sets that are indicative of specific immune cell types. For each sample, gene expression data are sorted in descending order based on the expression levels. The analysis computes enrichment scores by comparing the positions of genes within a set to their rankings in the expression data. In essence, gene sets that appear higher in the ranked list receive higher enrichment scores, suggesting greater representation. These scores provide estimates of the relative abundance of different immune cell types within the samples.

### Development of MAAGs risk score for sepsis

Univariate Cox analysis was carried out to explore the prognostic value of 480 DE-MAAGs for sepsis. Then, the feature prognostic variables were selected via LASSO analysis. Finally, according to the multivariate Cox analysis, the MAAGs risk score of each sepsis samples was evaluated as the formula: MAAGs risk score = CD160 × (− 1.73) + CX3CR1 × (− 1.51) + DENND2D × (− 2.01) + FAM43A × (− 1.49). “caret” package was performed to classify the sepsis samples into training and test cohorts with the partition criterion set at 6:4 and calculated the MAAGs risk score for each sepsis sample. On the basis on the optimal survival cutoff, the sepsis samples were divided into low- and high-risk groups.

### Establishment of nomogram and independent prognosis analysis

According to the clinical variables and 28 days survival outcome, a nomogram was established to evaluate the 7-, 14-, and 28 days survival probability for sepsis samples via “rms” script. “ggDCA” script was employed to evaluate the prediction power of nomogram, MAAGs risk score and clinical variables for sepsis. The independence evaluation of MAAGs risk score was carried out using univariate and multivariate Cox analysis. “survivalROC” script was carried out to estimate the AUC of 7-, 14-, and 28 days.

### Diagnostic effectiveness exploration and qRT-PCR analysis

GSE54514 (Daily samples for up to 5 days for sepsis survivors (n = 26), sepsis nonsurvivors (n = 9), and healthy controls (n = 18)) and GSE95233 (51 septic shock patients and 22 healthy volunteers were included in this study. Septic shock patients were sampled twice, at admission, and a second time at Day2 or Day3) datasets were used as an independent cohort to validate the diagnostic ability of CD160, CX3CR1, DENND2D and FAM43A for sepsis. “pROC” script was employed to calculate the AUC of CD160, CX3CR1, DENND2D and FAM43A in the training cohort (GSE65682) and test cohort (GSE54514 and GSE95233). A cohort of ten septic patients, diagnosed according to the Sepsis-3 criteria, was enrolled at Qilu Hospital with the approval of Qilu Hospital Ethics Committee. All research was performed in accordance with relevant guidelines/regulations, and that informed consent was obtained from all participants and/or their legal guardians. All research involving human research participants was performed in accordance with the Declaration of Helsinki. The selection criteria for these patients are detailed in Supplementary Table 2. A control group consisting of ten age-matched healthy individuals was also recruited from the same institution. Prior to their inclusion, all participants provided written informed consent. Blood samples (10 mL) were drawn from the septic patients within the first 24 h of their hospital admission, while those from the healthy controls were obtained at the time of enrollment. Total RNA was extracted from these samples using TRIzol reagent, and its purity and concentration were assessed with a NanoDrop 2000 ultraviolet–visible spectrophotometer (Thermo Scientific). Subsequently, the RNA was reverse transcribed to cDNA. Quantitative PCR reactions were carried out using the Bestar® SYBRGreen qPCR master mix (DBI Bioscience), and relative RNA expression levels were determined by the efficiency-corrected 2–ΔΔCT method, employing GAPDH as the internal reference^11^. Primer sequences for the target genes are provided in Supplementary Table 3.

### Statistical analysis

The data processing and analysis in this study were conducted in R language environment (R × 64 4.1.0) and GraphPad Prism (version 8.0.1). Correlations between the two components were calculated using Spearman’s correlation. Student’s t tests were used for statistical differences between two groups, and ANOVA tests were used between multiple groups. p.adjust < 0.05 was regarded statistically significant.

### Ethics approval

This human study was approved by the Ethics Committee of Qilu Hospital. All research was performed in accordance with relevant guidelines/regulations, and that informed consent was obtained from all participants and/or their legal guardians. All research involving human research participants was performed in accordance with the Declaration of Helsinki.

## Results

### Selection of DEGs and identification of macrophage-associated genes (MAAGs)

**Figure 1:**
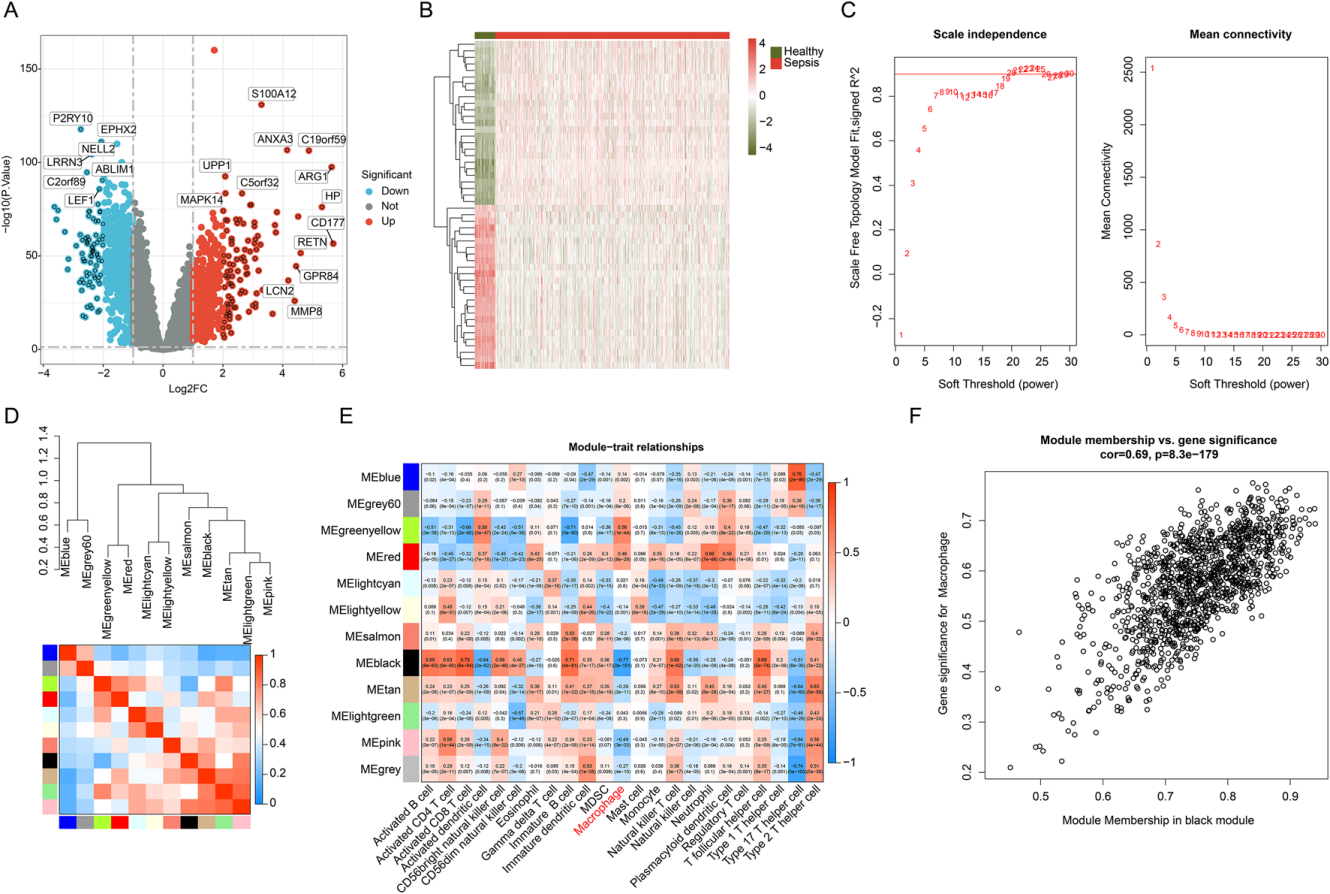
Differential expression analysis and WGCNA construction. (**A**) Analysis of DEGs between healthy and sepsis samples. Threshold for DEGs is set at |fold change| ≥ 2 and *p* value < 0.05. (**B**) Heatmap plot shows the top 20 up- and down-regulation DEGs. (**C**) Exploration of soft threshold. (**D**) Association analysis of each gene module. (**E**) Relationship of gene modules and immune cells. Blue color represents negative correlation and red color represents positive correlation. (**F**) Scatter plot reveals the correlation of module membership and gene significance for macrophage.

A total of 42 healthy samples and 479 sepsis samples were collected from the GSE65682 to determine the role of MAAGs for sepsis. Difference analysis was carried out to obtain the DEGs between healthy and sepsis groups based on the threshold set at |fold change|≥ 2 and *p* < 0.05 (Fig. 1A). The heatmap showed the top 20 DEGs in the healthy and sepsis groups (Fig. 1B). We subsequently developed a WGCNA to explore the crucial immune cell related genes for sepsis. The soft threshold was set at 19 to establish the scale free topology model (R^2^ > 0.85) and the mean connectivity revealed a good consistency in the scale-free network (Fig. 1C). Based on the dynamic tree, 11 gene modules were merged and the heatmap showed no clear association between each gene module (Fig. 1D). The result of module-trait relationships suggested that the gene modules were greatly related with immune cells, which the black module was the most characteristic module associated with macrophage (Fig. 1E). As displayed in Fig. 1F, the correlation of module membership in black module and gene significance for macrophage was 0.69 (*p* = 8.3e−179). Finally, the gene in the black module was considered as MAAGs and enrolled for the subsequent analysis.

### Identification of molecular subgroups for sepsis based on DE-MAAGs

**Figure 2:**
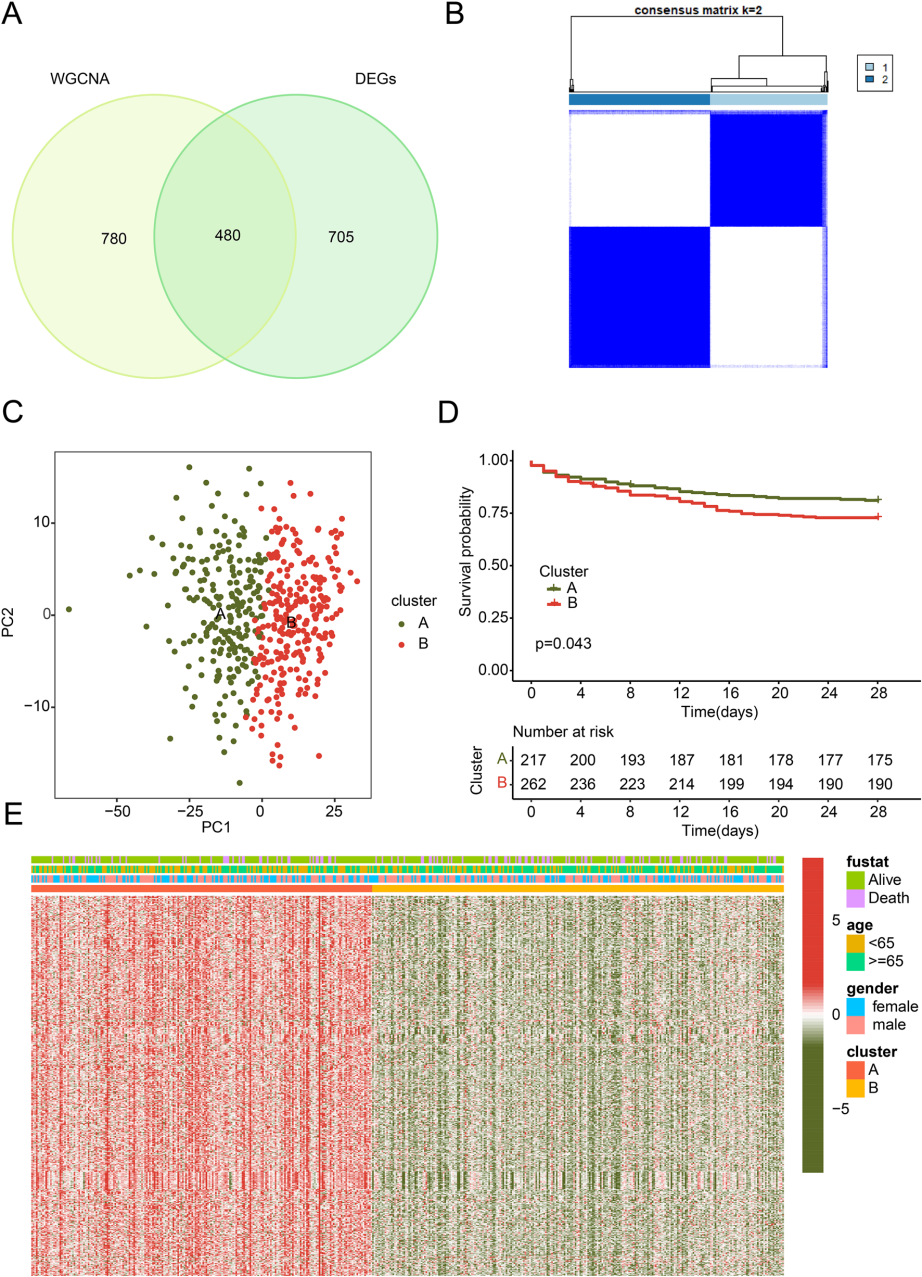
Molecular subgroup and clinical prognosis analysis for sepsis. (**A**) Generation of DE-MAAGs based on WGCNA and difference analysis. (**B**) Consensus clustering analysis. (**C**) PCA analysis based on DE-MAAGs. (**D**) 28 days clinical survival outcome of sepsis in cluster subgroups. (**E**) Relationship of DE-MAAGs expression and clinical features in cluster A and cluster B for sepsis.

The heterogeneity of clinical manifestations in sepsis is significant, and the evolution of the disease is influenced by numerous factors, which complicates personalized treatment. Through the utilization of weighted gene co-expression network analysis (WGCNA) and differential analysis, we collected a total of 480 intersecting differentially expressed MAAGs (DE-MAAGs). These were used to categorize sepsis samples into distinct molecular subgroups (Fig. 2A). Setting K = 2, we identified two molecular subtypes of sepsis: subtype A (containing 217 samples) and subtype B (with 262 samples) (Fig. 2B). Principal component analysis (PCA)-based differentiation, leveraging the DE-MAAGs, distinctly segregated samples within clusters A and B (Fig. 2C). Clinical survival outcome data indicated that the 28-day survival rate of sepsis samples from cluster A had a favorable prognosis compared to those from cluster B (*p* = 0.043, Fig. 2D). Further exploration of the association between DE-MAAGs and clinical characteristics in sepsis was undertaken. A heatmap depicted lower expression levels of the 480 DE-MAAGs in sepsis samples of cluster B, which had a poorer 28-day prognosis (Fig. 2E). These results demonstrate that DE-MAAGs can effectively ascribe sepsis samples to distinct molecular subgroups and correlate with 28-day clinical survival outcomes. Therefore, the use of MAAGs can effectively classify sepsis patients into two subgroups, laying the theoretical foundation for personalized treatment for sepsis patients.

### Function enrichment and immune infiltration assessment

**Figure 3:**
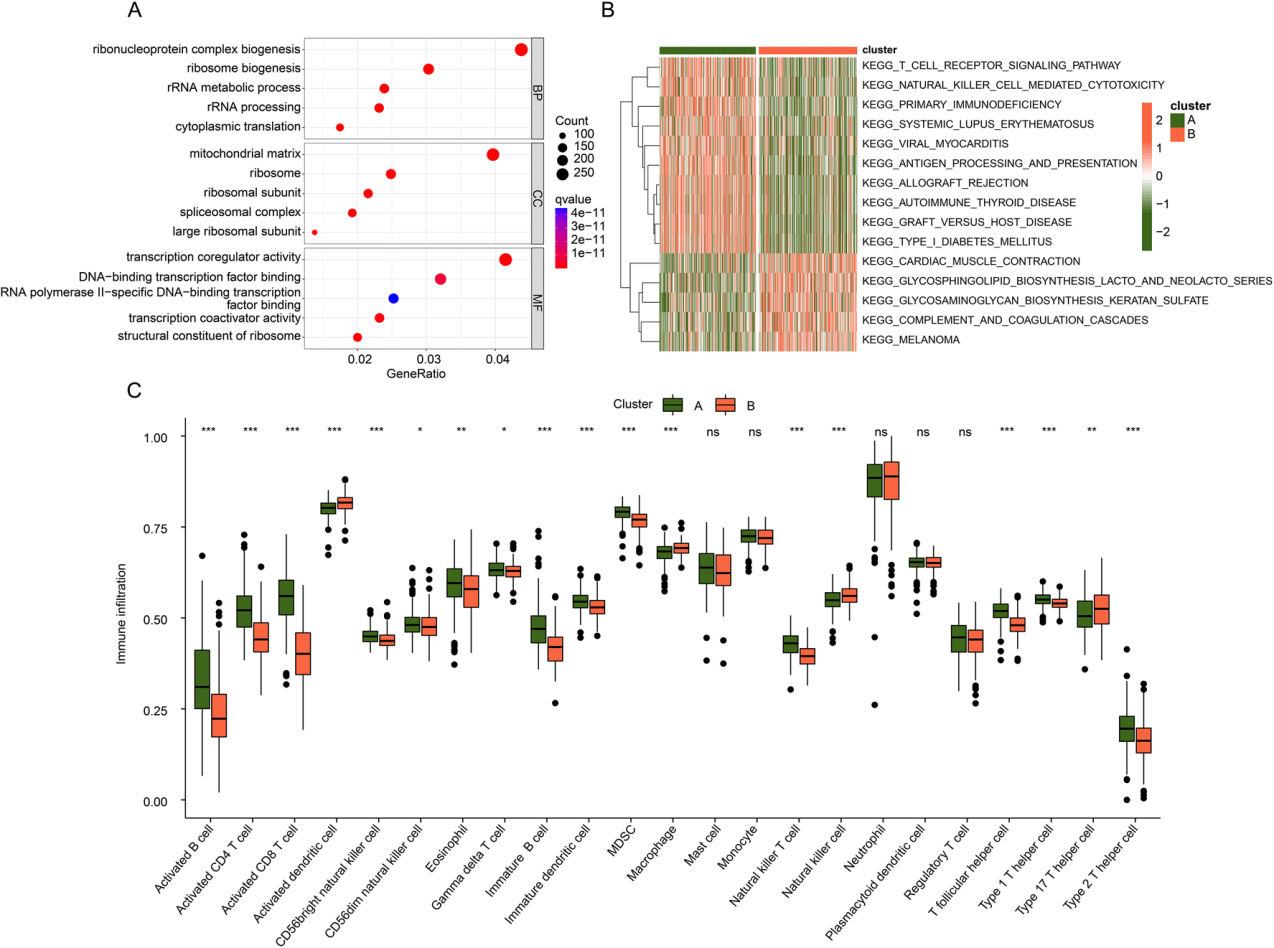
Exploration of function enrichment and immune infiltration assessment. (**A**) GO analysis of DEGs. (**B**) GSVA show the top 15 KEGG terms in DE-MAAGs based molecular subgroups. (**C**) immune infiltration estimation of 23 immune cells.

The immune infiltration of sepsis samples in DE-MAAGs-based molecular subgroups was further assessed. Under the selection threshold set at *p* < 0.05, the differential expression genes (DEGs) between DE-MAAGs subgroups were explored and the GO analysis implied that ribonucleoprotein complex biogenesis, mitochondrial matrix and transcription coregulator activity may mediate the function of DEGs in sepsis (Fig. 3A). GSVA result suggested that T cell receptor signaling pathway and natural killer cell mediated cytotoxicity were down-regulated of sepsis in cluster B (Fig. 3B). According to the estimation of ssGSEA, the fraction of 23 immune cells was assessed and the result illustrated the proportion of most immune cells was lower in cluster B, including activated B cell, CD4^+^ T cell and CD8^+^ T cell (Fig. 3C).

### Development of MAAGs prognostic signature based on the DE-MAAGs for sepsis

**Figure 4:**
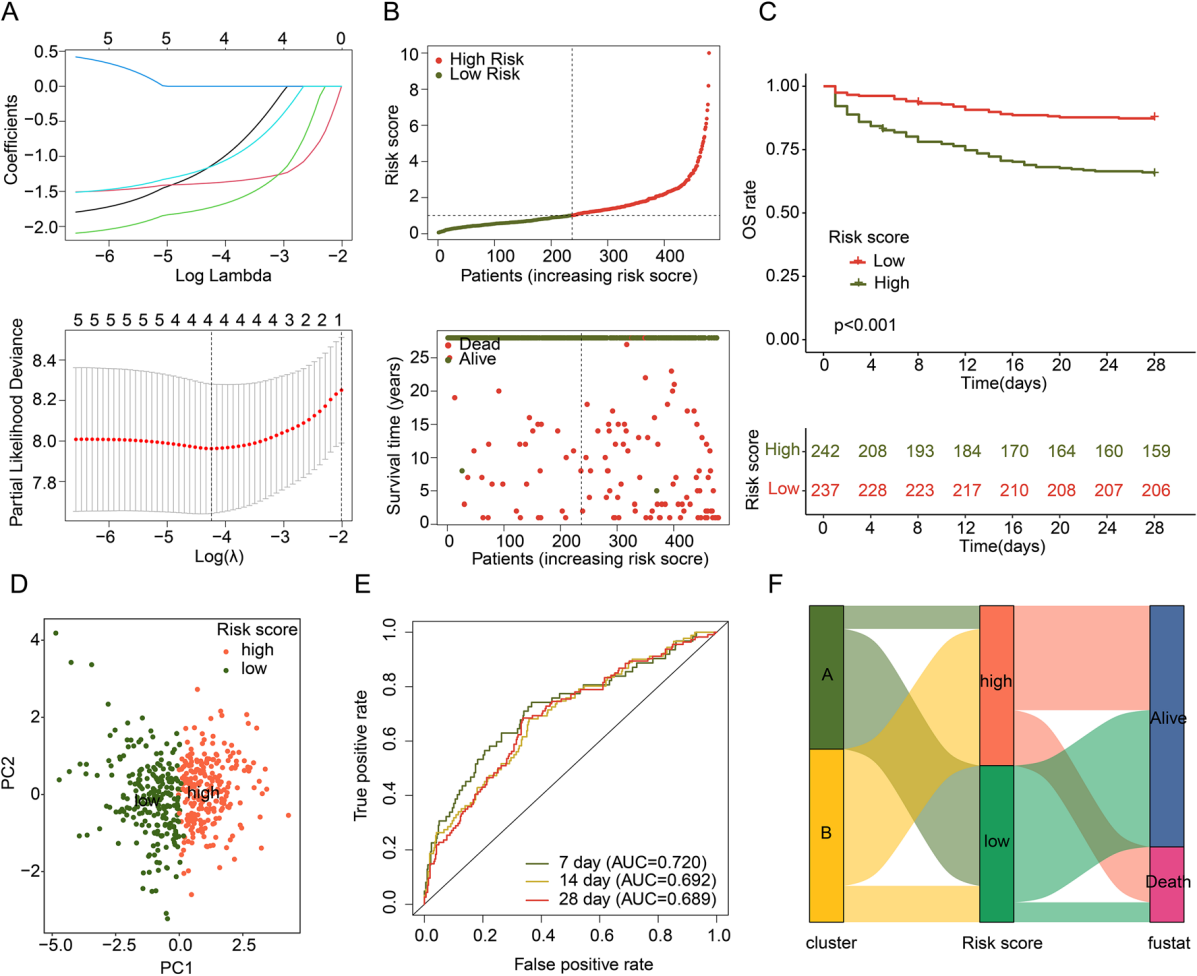
Development of risk model based on prognostic DE-MAAGs in sepsis. (**A**) Selection of feature prognostic DE-MAAGs. (**B**, **C**) Risk subgroups division and 28 days clinical survival outcome analysis. (**D**) PCA plot of sepsis in risk subgroups. (**E**) ROC analysis of 7-, 14-, and 28 days. (**F**) Schematic diagram of Sankey plot.

Based on the 28-day mortality and transcriptomic data of 479 sepsis patients, we developed a DE-MAAGs risk model for prognostic assessment. Based on the LASSO-univariate Cox analysis, 4 feature prognostic factors were obtained, including CD160, CX3CR1, DENND2D and FAM43A (Fig. 4A). On the basis of multivariate Cox analysis, the coefficient of 4 feature prognostic factors was estimated and the MAAGs risk score was evaluated for each sepsis sample. As exhibited in Fig. 4B,C, we discovered that the sepsis patients with low-risk score had better 28 days survival outcome (*p* < 0.001). The PCA scatter plot showed a clear distinction of sepsis between risk subgroups (Fig. 4D). The ROC result illustrated that the AUC of 7-, 14-, and 28 days was 0.720, 0.692 and 0.689, respectively (Fig. 4E). Sankey plot displayed the association of clinical survival status, MAAGs risk score and cluster subgroups for sepsis samples (Fig. 4F). We observed that most sepsis samples with poor prognosis in cluster B were preferred to be classified in the high-risk group and associated with worse prognosis.

### Validation of the MAAGs risk score

**Figure 5:**
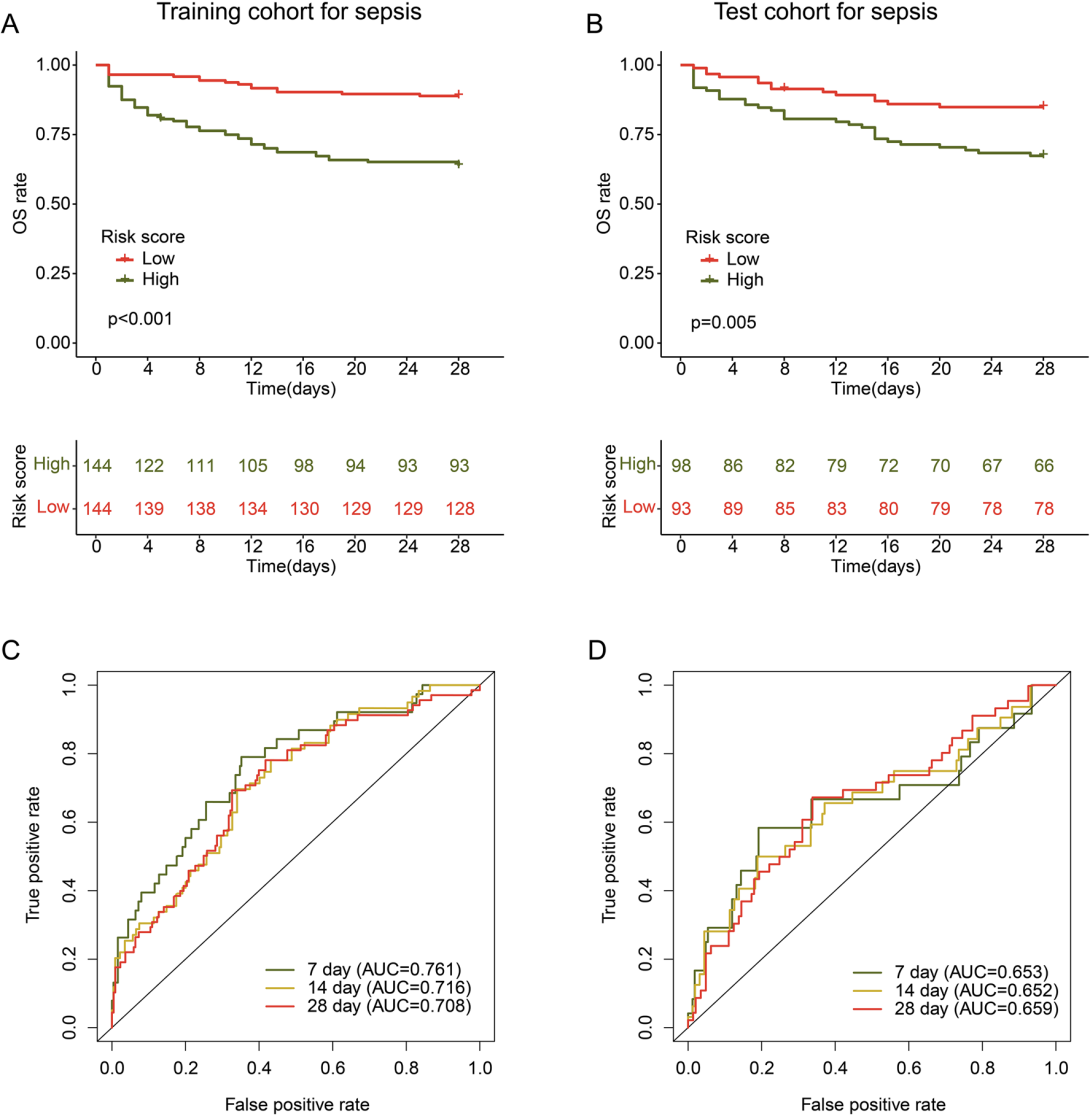
Verification of MAAGs risk score in training and test cohorts. (**A**, **B**) Analysis of 28 days clinical survival outcome for sepsis in the training and test cohorts. (**C**, **D**) Time-dependent ROC curve in the training and test cohorts.

The sepsis samples were divided into training cohort and test cohort to validate the independence of MAAGs risk score in predicting 28 days prognosis. As shown in Fig. 5A,B, the sepsis samples with high-risk score more tend to lower 28 days clinical survival outcome in the training and test cohorts. The ROC analysis indicated that the AUC of 7-, 14-, and 28 days was 0.761, 0.716 and 0.708 in training cohort, and 0.653, 0.652 and 0.659 in test cohort, respectively (Fig. 5C,D).

### Establishment of nomogram and independent prognosis analysis based on MAAGs risk score and clinical characteristic

**Figure 6:**
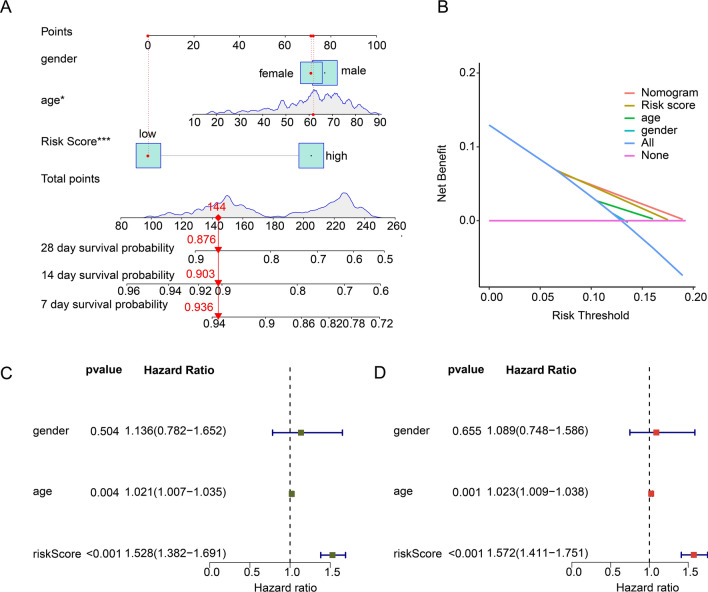
Nomogram construction and independent prognosis analysis based on MAAGs risk score and clinical features. (**A**) Nomogram construction of MAAGs risk score, age and gender. (**B**) DCA curve. (**C**, **D**) Independent prognosis analysis.

On the basis of MAAGs risk score and clinical characteristics, we established a nomogram to explore the 7-, 14-, and 28 days clinical survival probability for sepsis (Fig. 6A). The DCA result showed the ability of nomogram in evaluating survival probability for sepsis was superior to MAAGs risk score and clinical features (Fig. 6B). Moreover, the independent prognostic analysis revealed that the MAAGs risk score was an independent risk indicator for sepsis by univariate (*p* < 0.001, HR 1.528 (1.382–1.691)) and multivariate Cox analysis (*p* < 0.001, HR 1.572 (1.411–1.751), Fig. 6C,D).

### Evaluation of immune infiltration for sepsis in MAAGs risk subgroups

**Figure 7:**
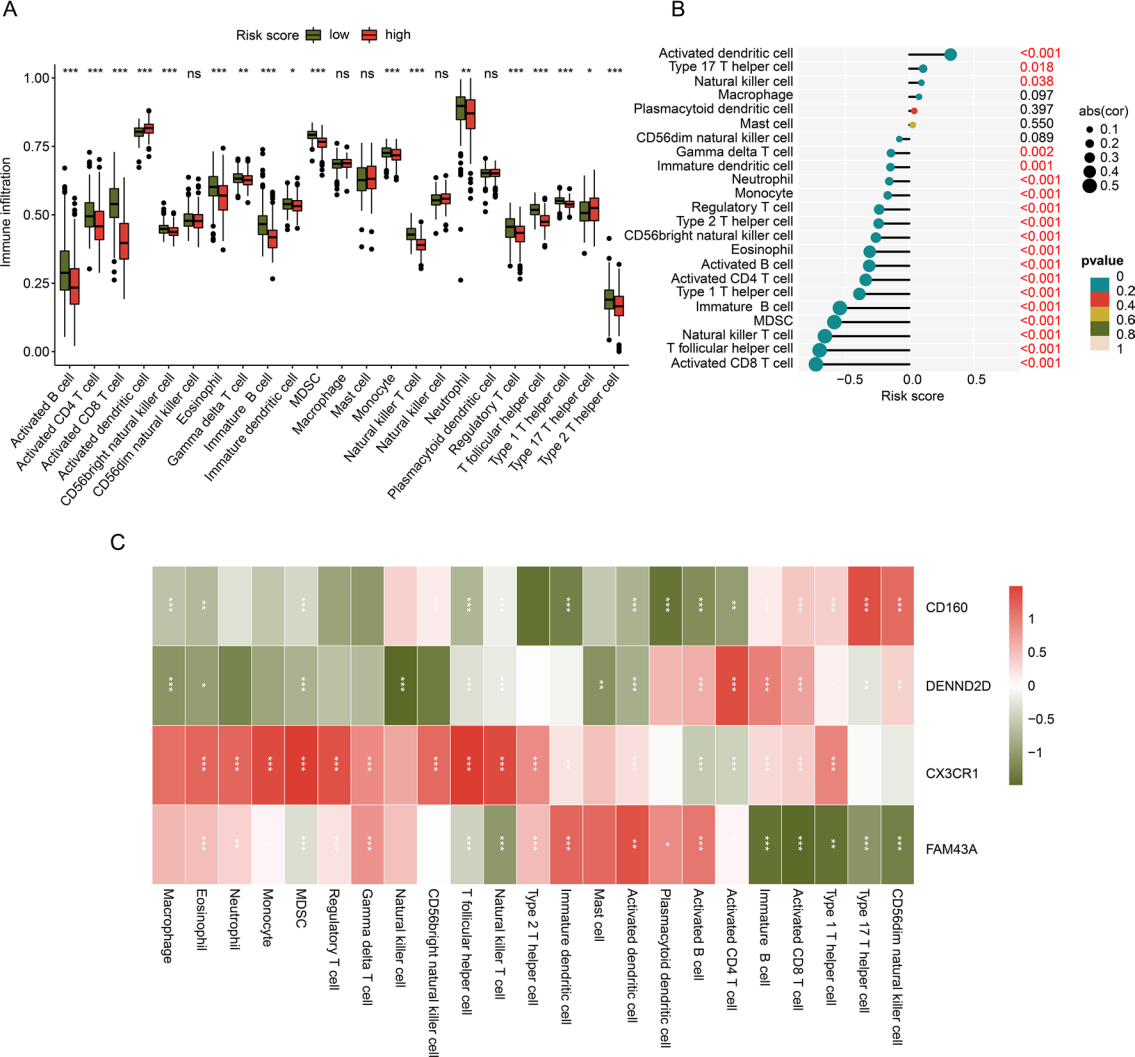
Association of MAAGs risk score and immune infiltration. (**A**) Immune infiltration landscape of sepsis in MAAGs risk subgroups. (**B**) Relationship between MAAGs risk score and 23 immune cells. (**C**) Correlation analysis of 4 prognostic factors and immune infiltration.

The immune infiltration of sepsis samples in MAAGs risk subgroups was further estimated via ssGSEA algorithm. As illustrated in Fig. 7A, we observed that the sepsis samples with low MAAGs risk score had higher proportion of most immune cells, such as eosinophil, immature B cell and natural killer T cell. Moreover, a notable association was observed of MAAGs risk score and immune infiltration, the MAAGs risk score was positively related with the portion of activated dendritic cell, type 17T helper cell and natural killer cell; but negatively related to other immune cells (Fig. 7B). The correlation result of 4 prognostic factors and immune infiltration subsequently revealed that CD160 was positively related to CD56dim natural killer cell, type 17T helper cell, type 1T helper cell, CD8^+^ T cell and immature B cell, but negatively related to CD4^+^ T cell, activated B cell and plasmacytoid dendritic cell; DENND2D was positively correlated with CD4^+^ T cell but negatively correlated with natural killer cell; CX3CR1 was positively relate to most immune cells; FAM43A was negatively correlated with type 1T helper cell, CD8^+^ T cell and immature B cell, but positively correlated with other immune cells (Fig. 7C). These results demonstrate that the MAAGs risk score is associated with immune infiltration and could reflect the immune status of sepsis samples in MAAGs risk subgroups.

### Diagnostic ability evaluation of 4 prognostic MAAGs for sepsis

**Figure 8:**
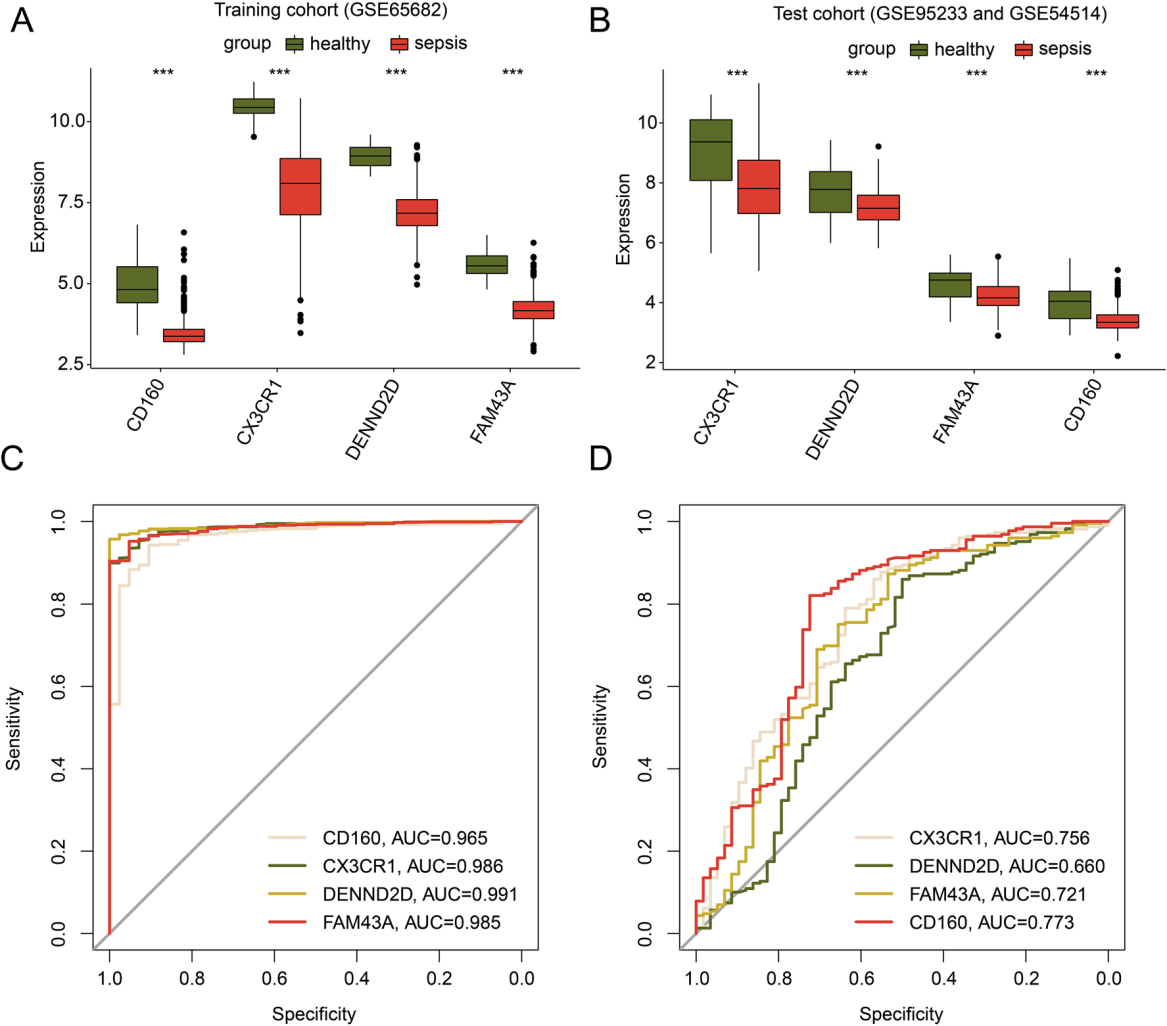
Exploration of diagnostic effectiveness for 4 prognostic DE-MAAGs. Expression profile of CD160, CX3CR1, DENND2D and FAM43A in (**A**) training cohort and (**B**) test cohort. (**C**, **D**) ROC analysis of CD160, CX3CR1, DENND2D and FAM43A in the training and test cohorts.

We further explored the diagnostic ability of CD160, CX3CR1, DENND2D and FAM43A for sepsis in both training cohort and test cohort. The difference analysis results suggested that the four genes are down-regulated in sepsis patients compared to controls in both training and test cohorts (Fig. 8A,B). The ROC curve results displayed that the AUCs of CD160, CX3CR1, DENND2D and FAM43A were 0.965, 0.986, 0.991 and 0.985 in the training cohort, and 0.773, 0.756, 0.660 and 0.721 in the test cohort, indicating a favorable diagnostic ability for sepsis (Fig. 8C,D).

### qRT-PCR analysis of 4 MAAGs in clinical samples

**Figure 9:**
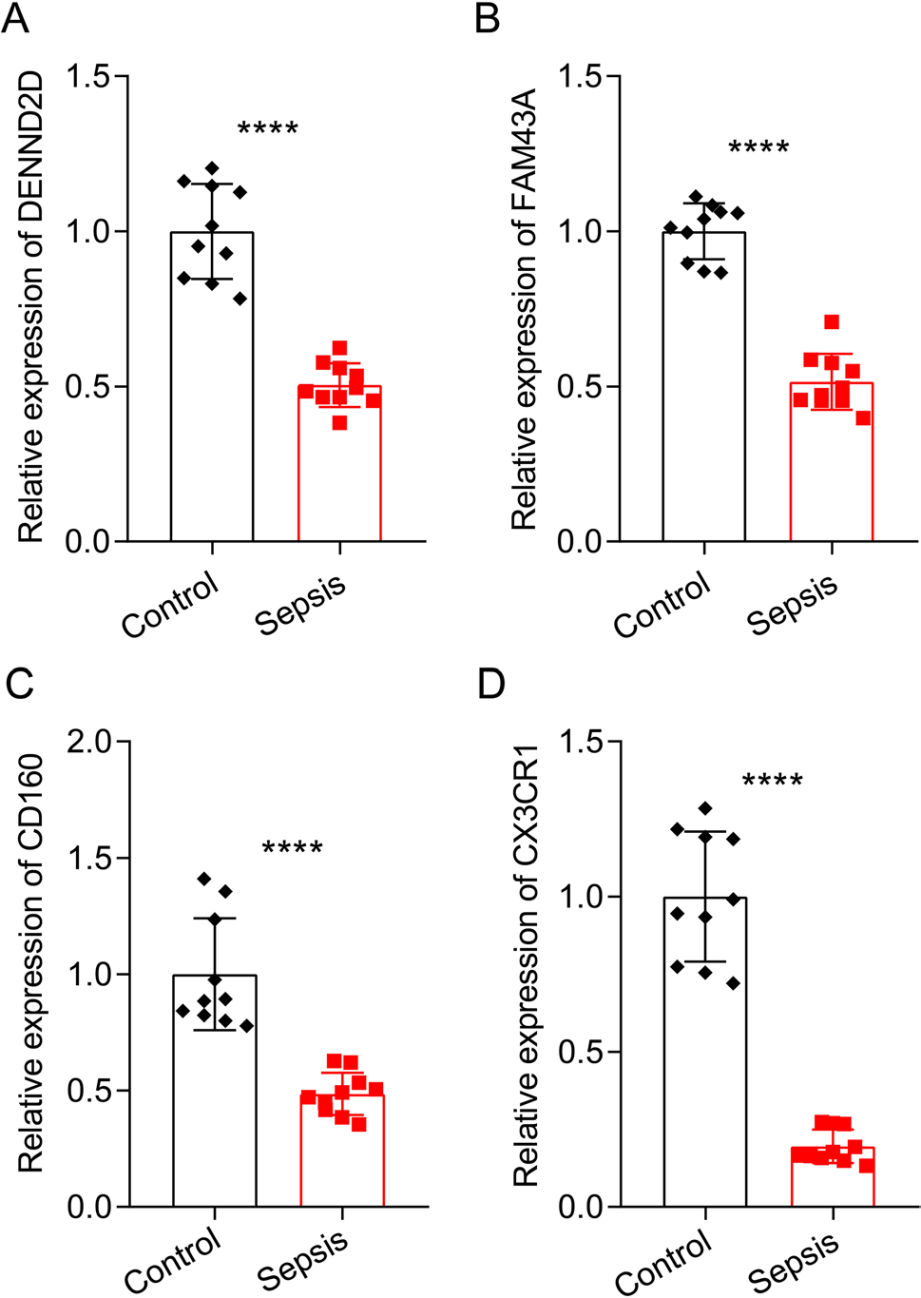
qRT-PCR analysis of 4 selected MAAGs in clinical samples. mRNA expression of (**A**) DENND2D, (**B**) FAM43A, (**C**) CD160 and (**D**) CX3CR1 in control and sepsis groups.

To further validate the diagnostic capability of the four genes CD160, CX3CR1, DENND2D, and FAM43A in clinical samples, we collected the 10 pairs clinical samples from the healthy and sepsis samples. As shown in Fig. 9, the qRT-PCR results exhibited that the mRNA expression of CD160, CX3CR1, DENND2D and FAM43A were obviously down-regulated in the sepsis group which was consisted with previous results.

## Discussion

Macrophages serve a dichotomous function in the evolution of sepsis. They are essential for phagocytosing necrotic cells and debris and synthesizing anti-inflammatory cytokines that mitigate pro-inflammatory reactions and facilitate tissue regeneration. Nevertheless, when macrophages become dysfunctional, they inadequately clear necrotic materials, thereby perpetuating inflammation and exacerbating sepsis^8^. Accordingly, a deeper investigation into MAAGs in the context of sepsis is imperative.

In this study, we identified four MAAGs as novel potential prognostic biomarkers and therapeutic targets in sepsis. CD160, a cell surface receptor predominantly expressed on natural killer (NK) cells and a subset of T cells, significantly contributes to the immune response by mediating cytotoxicity and cytokine production^12^. It exerts a protective effect in infectious contexts by boosting CD8+ T cell functionality and proliferation^13^. Although the precise role of CD160 in sepsis remains elusive, it has been implicated in fostering an amplified inflammatory response through its association with heightened cytokine secretion from NK cells, encompassing TNF-alpha, IFN-gamma, and IL-6^14^. Moreover, CD160 contributes to risk stratification by identifying sepsis patients at greater risk of poor outcomes and aids in selecting patient subgroups for trials of immunomodulatory drugs^15^. Our findings reveal a lower expression of CD160 in sepsis patients compared to controls, suggesting its utility as a negative prognostic indicator for sepsis. Given the recognized association between CD160 expression and CD8+ T cell exhaustion, the diminished CD160 levels in sepsis patients might reflect the detrimental hyperactivation of the immune system characteristic of sepsis progression^16^.

This study indicates that DENND2D is associated with the prognosis of sepsis. As a regulator of Rab GTPases and part of the DENND2 family^17^, DENND2D is implicated in the activation of the Rab signaling pathway, which is critical for intracellular communication^16,18^. Although there has been limited research on DENND2D specifically in sepsis, emerging evidence supports its involvement. For instance, Rab GTPases are known to modulate EGFR trafficking, thereby influencing macrophage activation during sepsis^19^. Given DENND2D’s role as a pivotal regulator of these GTPases, it could play a significant part in the pathophysiology of sepsis. Moreover, DENND2D serves as a tumor suppressor across several cancer types and disrupts MAPK signaling within tumor cells^20,21^. Notably, the MAPK pathway mitigates inflammation and mitochondrial damage associated with sepsis, suggesting a potential beneficial impact of DENND2D on sepsis outcomes^22^. Comprehensive research is required to delineate DENND2D’s function in sepsis more clearly and to assess its viability as a therapeutic target.

Unlike FAM43A, ample research exists on CX3CR1’s role in sepsis. CX3CR1’s interaction with its ligand, CX3CL1, has been acknowledged since 2008, where it was shown to facilitate the arrest and migration of pro-inflammatory cells during sepsis progression. The enhancement of inducible Nitric Oxide Synthase (iNOS)-mediated nitric oxide production and bactericidal pro-inflammatory cytokine production occurs via the NF-κB signaling pathway in septic peritonitis^23,24^. Additionally, reduced CX3CR1 mRNA expression levels have been identified as an independent biomarker in severe sepsis cases^25^. A clinical study involving 291 patients further indicated the potential of CX3CR1 as an early diagnostic biomarker for infections and sepsis in emergency settings^26^. Moreover, CX3CR1 plays a significant role in the immunomodulatory interaction between macrophages and the endothelium during sepsis, offering positive prognostic implications for affected individuals^27^. This array of findings aligns with the observed reduced expression of CX3CR1 in our cohorts of both sepsis patients and those at high risk for the condition, in contrast to their respective control groups. Our study additionally corroborates CX3CR1’s protective function in sepsis.

We observed a substantial decrease in eosinophil counts among high-risk sepsis patients as compared to their low-risk counterparts. Previous research largely supports the notion that diminished levels of eosinophils correlate with poorer sepsis outcomes^28,29^. The pivotal function of eosinophils in orchestrating immune and inflammatory responses both locally and systemically has garnered heightened scrutiny in recent years^30^. Given their relevance to the immune and inflammatory processes central to sepsis pathogenesis, eosinophils warrant further study. Historically, as early as 2008, eosinophils have been recognized for their ability to secrete mitochondrial DNA, a key mechanism in bacterial defense during sepsis^31^. Moreover, bacteremia survivors have exhibited increased eosinophil-to-neutrophil ratios^31^. Current hypotheses propose that a disbalance between type 1 and type 2 immune responses could precipitate severe sepsis symptoms^32,33^. Consequently, eosinophils might confer a protective effect in sepsis through their involvement in type 2 immunity^34^. The potential of harnessing type 2 immune activation via eosinophils in severe infections presents a promising avenue for therapeutic intervention to mitigate the deadly inflammatory cascade in sepsis, necessitating further scholarly exploration^34^.

This study possesses certain limitations, as it primarily focuses on the analysis of public databases. The investigation recognizes potential bias stemming from its confinement to a single region, which is a notable constraint when compared to multi-centric clinical research endeavors. Although the research identified multiple aberrantly expressed genes (MAAGs) that correlate with prognostic outcomes in sepsis, the exact mechanisms by which these genes impact patient survival warrant more comprehensive investigation. Moreover, our study differentiated sepsis patients into two subgroups via consensus clustering analysis, uncovering substantial variances in survival rates and immune microenvironment characteristics. This differentiation provides a foundation for tailored and precise therapeutic strategies for sepsis, yet these insights call for further clinical corroboration. In conclusion, while our study reveals novel perspectives on the management of sepsis, there remains a significant amount of work to be done to refine these insights for practical clinical applications.

In conclusion, our study successfully developed an efficacious prognostic model for sepsis risk stratification, utilizing four MAAGs. While the investigation into the underlying mechanisms of this model was limited by the absence of in-depth in vivo or in vitro experiments, our findings contribute fresh insights into the function of MAAGs in sepsis and identify novel potential targets. This research offers a novel foundation for clinical sepsis risk stratification and suggests avenues for more tailored and precise treatments in the future.

### Supplementary Information

Supplementary Table 1.

Supplementary Table 2.

Supplementary Table 3.

## Data Availability

The datasets (GSE54514, GSE65682 and GSE95233) for healthy and sepsis samples were acquired from the Gene Expression Omnibus (GEO) database (https://www.ncbi.nlm.nih.gov/geo/) in this study.
