# Supplementary material for: Development of macrophage-associated genes prognostic signature predicts clinical outcome and immune infiltration for sepsis
Source: Sci Rep. 2024 Jan 23;14:2026. doi: 10.1038/s41598-024-51536-3 (PMC10805801; doi:10.1038/s41598-024-51536-3)
Supplement: Supplementary file 2 — Supplementary Table 2. [file 41598_2024_51536_MOESM2_ESM.docx]

**S****upplementary Table 2:** Inclusion and Exclusion Criteria for Sepsis Patient Selection.

| Inclusion criteria | 1. Confirmed diagnosis of sepsis. 2. Patient has provided informed consent and has agreed to participate on a voluntary basis. 3. Age range between 18 and 80 years, inclusive of 80 years old. 4. Documented general health status. |
| --- | --- |
| Exclusion criteria | 1. Individuals aged greater than 80 years or equal to or less than 18 years. 2. A documented history of chronic cardiac, hepatic, or renal disorders. 3. Women who are currently pregnant or breastfeeding. 4. Presence of hyperlipidemia, diabetes mellitus, or other metabolic conditions. 5. Concomitant severe primary diseases pertaining to the cardiovascular system, liver, kidney, or hematopoietic system. 6. Co-occurrence of psychiatric illnesses. 7. A history of prolonged use of sedatives or habitual alcohol consumption. 8. Concurrent diagnoses of malignancies or immune deficiencies, or other conditions significantly affecting immune function. 9. Declination by the patient or their immediate family to participate in the research study. |
