# Supplementary material for: Development of macrophage-associated genes prognostic signature predicts clinical outcome and immune infiltration for sepsis
Source: Sci Rep. 2024 Jan 23;14:2026. doi: 10.1038/s41598-024-51536-3 (PMC10805801; doi:10.1038/s41598-024-51536-3)
Supplement: Supplementary file 3 — Supplementary Table 3. [file 41598_2024_51536_MOESM3_ESM.docx]

**Supplementary Table 3:** qRT-PCR primer sequences of target genes

| **Genes** | **Forward primers** | **Reverse primers** |
| --- | --- | --- |
| *CD160* | *5’-* *ACAGACTACAACTGCCCAGC-3’* | *5’-* *TATCCTGCCCACTCTCTGCT-3’* |
| *CX3CR1* | *5’-* *TGGCCAAACACTGAGACCAA-3’* | *5’-* *GTGAAGGCCTCTAGTCGCTG-3’* |
| *DENND2D* | *5’-* *CCCCGAACCTGTCCCCTA-3’* | *5’-* *CTTCAGTCTCAGGCTGGCTC-3’* |
| *FAM43A* | *5’-* *CTGTGAGCCTGCACTACTCG-3’* | *5’-* *AGAGCACGGTGTAAGTTGGG-3’* |
| *GAPDH* | *5’-* *AATGGGCAGCCGTTAGGAAA-3’* | *5’-* *GCGCCCAATACGACCAAATC-3’* |
